# Supplementary figures and images for: The Search for an Interesting Partner to Combine with PD-L1 Blockade in Mesothelioma: Focus on TIM-3 and LAG-3
Source: Cancers (Basel). 2021 Jan 14;13(2):282. doi: 10.3390/cancers13020282 (PMC7838786; doi:10.3390/cancers13020282)

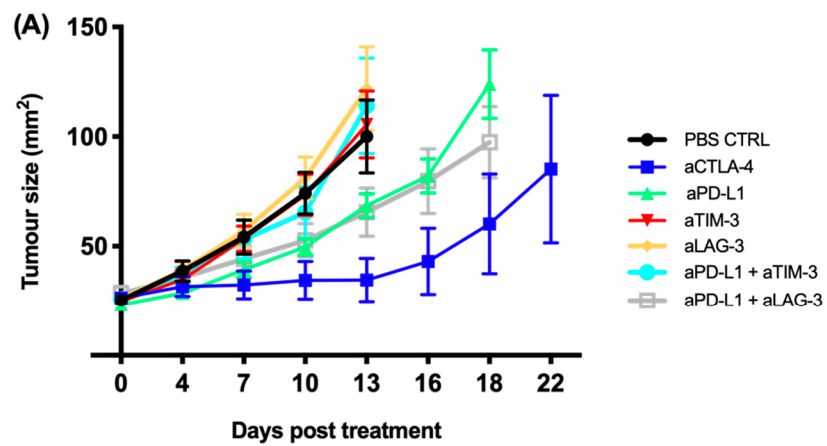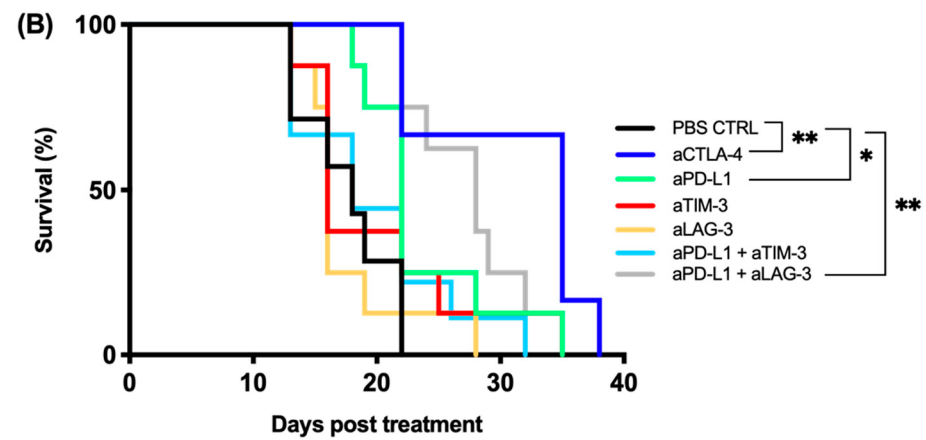

Supplement: Supplementary file 1 [file cancers-13-00282-s001.pdf]
